# Fractal analysis of palmar electronographic images. Medical anthropological perspectives

**Published:** 2008-11-15

**Authors:** Guja Cornelia, V. Voinea, Baciu Adina, M. Ciuhuţa, A. Crişan Daniela

**Affiliations:** * „Francisc. I. Rainer” Anthropological Institute of the Romanian Academy, Bucharest, Romania; ** Department of Automatic Control and Computer Science, University Politehnica of Bucharest, Bucharest, Romania; *** Romanian American University, Bucharest, Romania

**Keywords:** health-adaptability-illness, structure-shape-function, spectrum of fractal dimension, adaptive interface

## Abstract

*The present paper brings to the medical specialists’ attention a possibility of multivalent imagistic investigation – the **palmar electrographic method** submitted to a totally new analysis by the fractal method. Its support for information recording is the radiosensitive film. This makes it resemble the radiological investigation, which opened the way of correlating **the shape** of certain structures of the organism with their function. By the specific electromagnetic impressing of the ultra photosensitive film, **palmar electrography** has the advantage of catching **the shape of certain radiative phenomena**, generated by certain structures in their functional dynamics – at the level of the human palmar tegument. This makes it resemble the EEG, EKG and EMG investigations. The purpose of this presentation is to highlight a new modality of studying the states of the human organism in its permanent adaptation to the living environment, using a new anthropological, informational vision – by fractal processing and by **the couple of concepts system / interface** – much closer to reality than the present systemic thinking. The human palm, which has a special medical-anthropological relevance, is analysed as a complex adaptive biological and socio-cultural interface between the internal and external environment. The fractal phenomena recorded on the image are ubicuitary in nature and especially in the living world [**1**,**2**,**3**,**4**] and their shapes may be described mathematically and used for decoding their informational laws. They may have very usefulimplications in the medical act. The paper presents a few introductory elements to the fractal theory, and, in the final part, the pursued objectives are concretely shown by grouping the EG images according to certain more important medical-anthropological themes*.

## Introduction

**Fig. 1:**
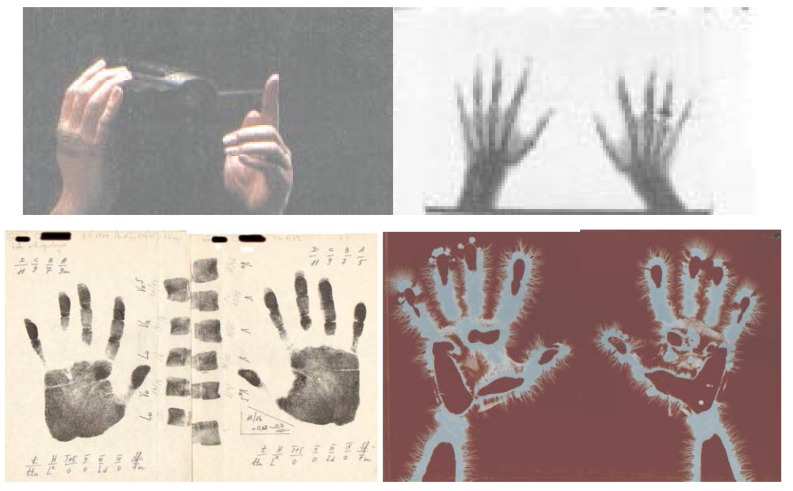
Four categories of investigation of the upper limb extremities: a) photographic image, b) radiographic image, c) dermatoglyphs (palmar prints), d) electrographic image (electrographic prints).

It is well known that, besides the brain, ***the hand*** is the most important organ for maintaining life in the conditions specific of the human species. Next to the face, it has the greatest individual expressivity. It is a symbol of the individual’s uniqueness, offering complete, valuable information regarding man as a species and as an individual. The hand is a hypercomplex interface between the organism and the specific living environment, between the biological and the social, between the present and the past of populations, between the normal and the pathological. Taking under consideration all of these, and especially the importance of palms for man’s evolution, the preoccupations regarding their study during the history of knowledge are natural, very interesting and useful. The hands have been the object of study for many fields, mainly the behaviour sciences, medicine, anthropology etc.(**Fig. 1 a,b,c,d**).

**Fig. 2:**
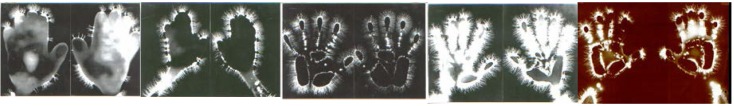
Types of electrographic images from left to right: dielectric, hydric, semiconductor, mineral, mixed

**Fig. 3:**
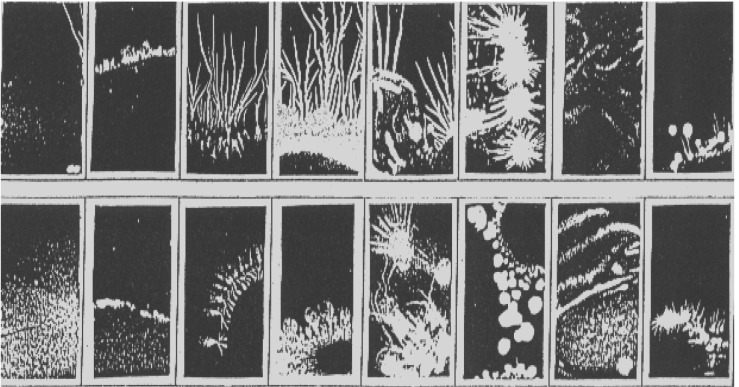
Types of streamers (electrical discharges) in electrographic images from left to right, top: anodic, bottom: cathodic: 0, 1, 2, 3, X, Y, Z, I

For about three decades the EG images [**5**,**6**,] were analysed subjectively, in the same way as radiological images. For the detailed examination of the radiographic image the following are appreciated: *width, shape, structure, positioning, outline and its intensity, the fact that images appear isolated or in groups, distinct or blurred contours correlated with the functional state of the examined part* [**7**]. In order to examine electrographic images, we have in view the following characteristics: density of electric discharges (streamers) on the palm outline, their shape, size, positioning, right/left symmetry, their polarity: **Figures 1**, **2**, **3**.

The analysis of these signals on the human palms and soles, which reflect the shape of the electromagnetic field (em.) in the functional dynamics of the entire organism, may be correlated with the designed morphoanatomical structure and function, associated or correlated with the investigated interface. The EG image in **Fig. 1d**. represents palmar electrographic images of a human object in normal, every day state. We easily recognize the palms with discrete dermatoglyphic prints alongside with a great diversity of streamers placed on the outline and on the surface. They are distributed with different densities, shapes, intensities and sizes and a fractal structuring, on levels, at finger extremities. The complexity of streamer configurations reflects the electrophysiological dynamics of the human palmar tegument and the active part of mediator and multiple psychoneuroendocrine interface of the organ skin. They describe the informational bioelectromagnetic interaction [**3**,**4**,**6**,**8**] of the human organism with the environment. They suggest the characteristics of a mineralized substratum associated with the type of bioelectric mineral (**Fig. 2**), characterized by domination of streamer ‘3’ (**Fig. 3**).

In this way we bring to the medical specialists’ attention an aspect of a complex phenomenon of reaction and adaptation to the environment having great dynamism: the permanently fluctuating form that accompanies the vital activity of the organism, that can be revealed at the level of the body surface by means of electromagnetic fields (em.). Part of them have been studied and are still being studied by means of electromagnetic recording methods of variations of the electrographic signal in case of EEG, EKG, and EMG investigations.

The **purpose** of this paper is to place at the medical specialists’ disposal electrographic recordings (EG) of the palms, significant both for anthropology and for medical practice, applying fractal analysis in order to decode the obtained information and to interpret it. Palmar electrography has a unique specificity: an electrical discharge occurs, it impresses the radiological film and brings new data on the shape and structure of the em. fields around our body. These fields accompany and are coupled with the biochemical substratum of the human organism. Today their shapes may be mathematically described , may be appreciated in point of quantity and may ‘measure’ the investigated phenomena. This helps to better understand the complexity of the human being and of its interaction with sofisticated, elevated modalities of adaptation within the context of the great environmental diversity. *We started from the hypothesis that the EG image catches aspects of variability of physiological and psychological states of the organism and its reactivity at tegument* level by the radiating fields existing in the proximity of the human body (caloric radiations, as well as other radiations of the em. spectrum). In this way, there is the possibility of **appreciating the variability and the reactivity degree** of the organism by means of palmar interface, as a measure of its adaptability – of the modifications as a response to the action of environmental factors and to the capacity of going back to the initial form after the factors cease to exist. At the same time these investigations evaluate the oscillations of health condition (adaptability, normality) or illness condition (lack of adaptation, abnormality), highlighting the **degree of adaptability**. It shows the diversity of informational integrating modalities man has in the living environment. *The detailed examination of EG with a view to establishing an electrographic diagnosis includes: appreciation of the bielectric type (**Figure 2**) and of the streamer kind (**Figure 3**), of the state of electrical equilibrium, of symmetry: right/left, of the bioelectrical polarization: negative/positive (cathodic/anodic) localization of palmar or plantar areas with peculiar aspects, and, finally, establishing a diagnosis of system and organ.*

## Materials and methods

The images in the present paper have been selected out of a great number of EG palmar recordings resulting from researches carried out over three decades in the Individual’s Anthropology Laboratory – electrographic explorations at „Fr. I. Rainer” Anthropological Institute of the Romanian Academy. Plantar and front or simultaneously palmar-plantar EG may also be obtained (Dr. C. Guja’s Electrotheque).

The ***methods used for analysis*** were: comparison, analogy and complementarity of em. signals as well as similarity of EG signals, the images being processed later by means of the computer.

The **objectives** pursued in order to analyse the results are to be found in grouping the EG images according to four themes, which open new directions for study in medicine and anthropology:

I. The first direction refers to the possibility and advantages of dynamic, comparative ***investigation in time***, (longitudinal research) of the same subject – the EG method being non-invasive, cheap –for instance images taken before, during and after various therapies (with medicines, electrotherapy, phototherapy etc.) [**9**].

II. The second direction is given by the qualities of the method, which is ***sensitive to immediate, spontaneous physiological changes*** of the human organism: for instance monitoring of reactivity and recovery time, returning to equilibrium of the organism in case of various solicitations, stimuli, stress etc.

III. The third direction is given by the possibility to ***highlight sensing of stimuli*** by the human organism by means of unknown communication modalities, probably complementary to those already known: for instance, the ways of sensing the *sound by the deaf and dumb or those of sensing the light by the blind*.

IV. The fourth direction refers to variability of EG images depending on the medical diagnosis to the possibility of an EG medical-anthropological diagnosis (interface diagnosis) [**8**]. To finding new informational diseases [**10**,**11**] based on the analysis of the spectrum of fractal dimension and the comparative study of the average fractal dimension, too.

**The research electrographic technique** employed is original [**5**,**6**], adequate to the study of the radiative fields of bodies. This enables testing of the human body electromagnetic fields submitted to a field generated by a unique, monopolar, positive/negative, high-voltage (4-35lV) and low intensity (50mA) signal. The signal has a triangular shape, with sudden ascending slope (15-30 microseconds) and slow descendent slope (100-500 microseconds). The analysed images were recorded on radiological films, placed on the glass screen of the electrograph, on which the subjects under study also placed their palms, by successive start of the positive/negative signals on the same film or on different films.

The fractal method, by *analysing the spectrum of the fractal dimensions* and by the average fractal dimension of these EG recordings, offers a long expected opportunity, very useful for automatic processing.

**Fig. 4a,b:**
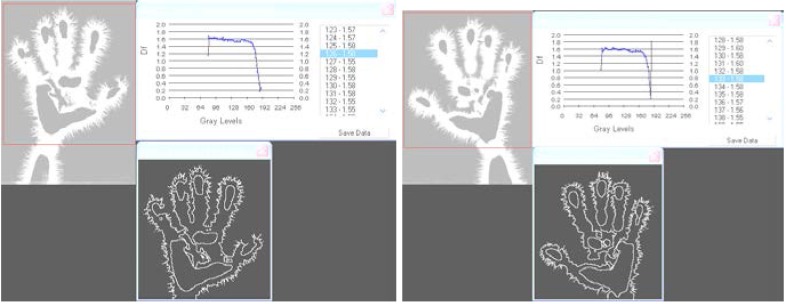
EG image of the left **(4a)**/right **(4b)** hands processed by means of fractal method – analysis of fractal dimension spectrum.

***The calculation of fractal dimension, „box-counting”algorithm***. Fractals are objects with irregular contours, self-similar (they get repeated) while entering its details, at any examining scale. ***It follows that, actually, any component of the living organism may be treated as a fractal***. Mathematician Mandelbrot [**1**] studied in the sixties the complex irregular shapes in nature, which he called fractals. He laid the basis of fractal, non-Euclidean geometry. After fractal geometry was founded, scientific knowledge got back to real world. The geometry we are familiar with, Euclidean geometry, has accustomed us with regular, ideal, abstract shapes: the line, the straight line, the plane surface, regular volumes. It is obvious that the shapes of rivers, mountains, even that of the Earth, in all details, as well as all living forms are of fractal type.

**Fig. 5:**
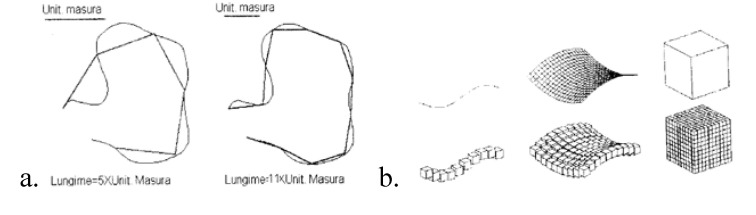
**a.** Dependence of a curve length on the employed measuring unit. **5b.** Covering of three Euclidean figures with cubes having equal sides.

A defining trait of fractal objects is dependence of their size on the measuring unit used (**Fig. 5a**). If one chooses a finer measuring unit, an irregular outline is easier to express, with greater accuracy, also highlighting its details (the smaller unit detects more details of an outline and a more precise measurement is taken).

**When the size depends on the employed scale, fractal objects are more difficult to measure within the context of classical, Euclidean geometry**. *Euclidean dimension D* is the number of coordinates required to define any point of the object, or more precisely, the dimension of the Euclidean space in which the analysed object may be introduced: the straight line in the plane, the cone in the tridimensional space.

*Topological dimension T* is defined by local properties of the points of the object under analysis and corresponds to the notion by which a point is of size 0, a line or a thin curve are size 1, surfaces are size 2, volumes are of size 3 etc., without taking into account the larger size, of the Euclidean space in which these forms are plunged.

When fractals appeared, characterization of a shape by its topological dimension, expressed by an integer, proves to be insufficient. In this way the notion of fractal dimension Df expressed by a rational number is introduced.

The German mathematician Felix Hausdorff defines a new concept regarding topological spaces, suggesting in this way that **fractal dimension is proportional to the minimum number of spheres, with a given radius required to cover the measured object**. For easier work on the computer, for covering we considered cubes instead of spheres. For instance, in order to cover a curve of length 1 it is necessary to have N(s)=1/s cubes of side s (**Fig. 5b**). In order to cover a surface of area 1 it is necessary to have N(s)=1/s2 cubes of side s, and finally, in order to cover a cube of volume 1 it is necessary to have N(s)=1/s3 cubes of side s, and inductively the relation: N(s)~1/sD is checked, where N(s) is the number of cubes of side s; s is the scale coefficient or the length of the covering cube side; D is the object dimension. 

Using the logarithm for the relation above, we can infer D: D ≈ log(N(s))/log(1/s).

Hausdorff dimension is accepted as a good approximation of fractal dimension Df, and according to this **hypothesis**, fractals are shapes whose Hausdorff dimension strictly exceeds the topological dimension.

**Yet, fractal dimension is difficult to calculate. There are calculation algorithms for** fractal dimensions, and one of the easily implemented algorithms is box-counting algorithm [**13**]. In specialized literature there are many attempts to assess fractal dimension [**14**,**15**,**16**,**17**]. The algorithm based on the „box-counting” method has two major advantages: it is easily implemented when the computer is used and may be applied for images of great complexity. „Box counting” fractal dimension derived from Hausdorff covering dimension is therefore given by the approximate relation:

$$D\approx \frac{\log\left(N(s)\right)}{\log\left(1/s\right)}$$

One expects that for as small an s as possible, the above approximation is as good as possible:

$$D=\underset{s\underset{}{\to }0}{\lim}\log\frac{N(s)}{\log(1/s)}$$

If this limit exists, it is called „box-counting” dimension of the measured object. As in practice this limit is slowly convergent, an alternative way is employed. As expression:

$$\log(N(s))=D\cdot \log\left(\frac{1}{s}\right)$$

is the equation of a straight line of slope D, the curve „log-log” is drawn, described by the coordinating points (log(N(s), log(1/s)). By linear regression (the method of smallest squares) one determines the slope of the straight line which approximates dots distribution, this being the sought fractal dimension. The regression straight line has the form: Y=a⋅X+band the straight line slope (the value of coefficient „a”) represents fractal dimension.

„Box-counting” algorithm implies determination of fractal dimension according to object outline dependence on the scale factor employed. It consists in successive covering of the image with squares having equal sides (2, 4, 8…) and counting, each time, the squares that cover the object outline. Coordinating points *(log(N(s), log(1/s))* where s is the common side of the covering squares and N(s) is the number of squares containing information, will be placed approximately along a line, and its slope will be the fractal dimension in “box-counting” sense. In a synthetic representation, the algorithm determining “box-counting” dimension for binary images is the following: 

*1. the original image (binary) is read*

*2. the analysis area is selected*

3. box-counting dimension is calculated, counting each time number N(s) which contains at least one dot of the shape. The obtained values are calculated with logarithms and graphically represented in a curve whose slope is box-counting dimension.

As the electronographic images are grey, an extended procedure was used; for each level of grey of the selected image, fractal dimension is calculated using the algorithm described before. Finally, the fractal dimension spectrum of the selected image is obtained.

## Results and discussions

**EG images taken at three distant time intervals, selected out of about 1700 recordings of subject L.G., investigated over 30 years : 1975-2008, studied longitudinally – **Fig. 6 a**, **b**, **c**.**

**Fig. 6a:**
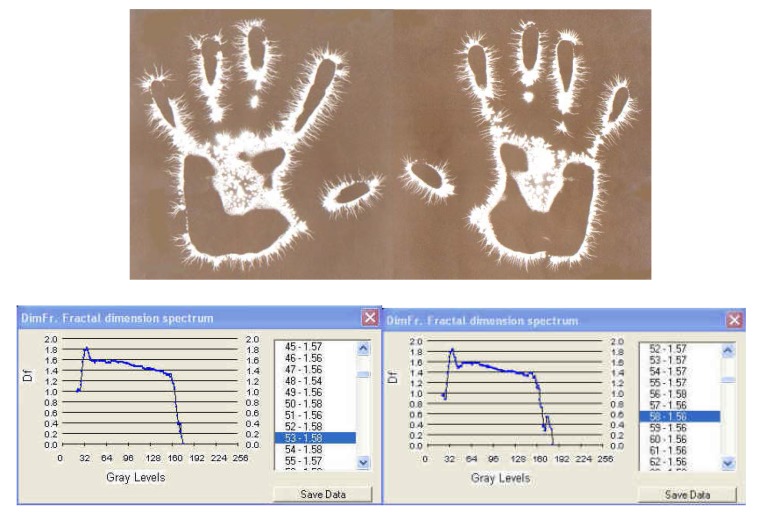
EG images with fractal dimension spectrum of both hands – 21 March, **1978**, time: 8:30 – subject: L.G. One can see ramified luminescences along the palmar outline, topographically marking certain areas whose asymmetry is closely followed by the fractal diagrams.

**Fig. 6b:**
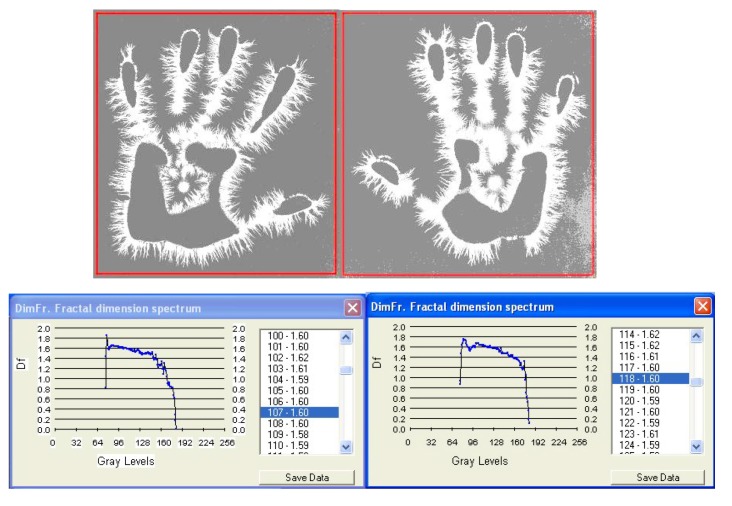
EG images with fractal dimension spectrum of both hands taken on 7 July, **1984**, time: 8:30-subject L.G., ***after an interval of six years from the initial investigation***.

**Fig. 6c:**
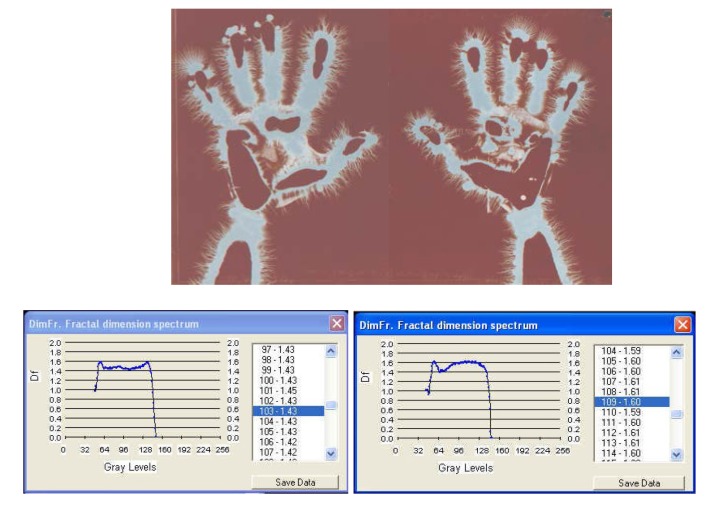
EG images with fractal dimension spectrum of both hands taken on 1 February, **2008**, time:12:25, ***after an interval of 30 years from the initial investigation***.

**EG images taken at three different moments in time: before and after generating a sharp acoustic stimulus**.

**Fig. 7a:**
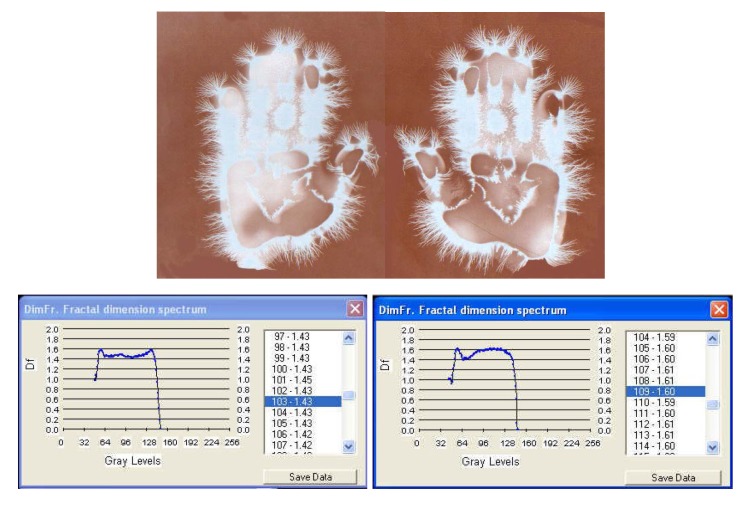
EG images with fractal dimension spectrum of both hands of subject L.G. – ***witness sample for sharp acoustic stimulus***

**Fig. 7b:**
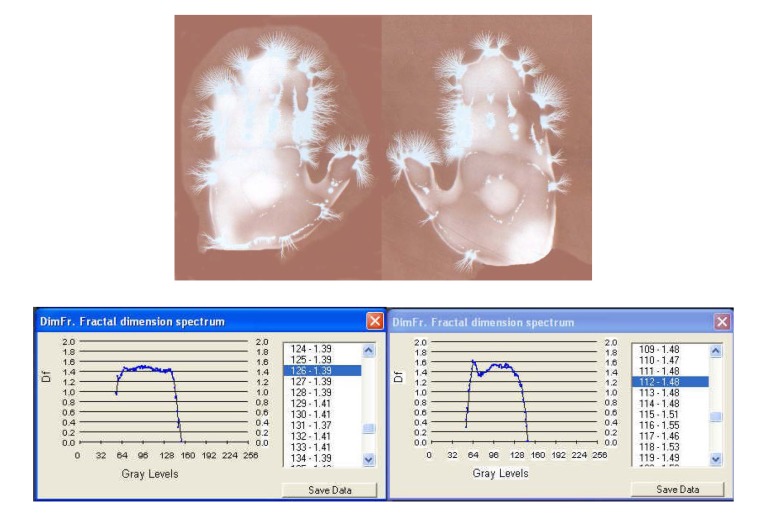
EG images with fractal dimension spectrum of both hands of subject L.G. immediately after a sharp whistling – ***30 seconds***.

**Fig. 7c:**
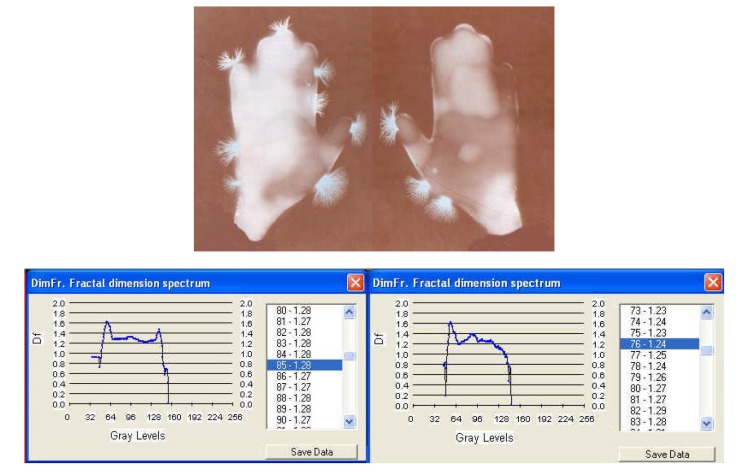
EG images with fractal dimension spectrum of both hands of subject L.G. immediately after a sharp whistling– ***30 seconds***

**Fig. 8a:**
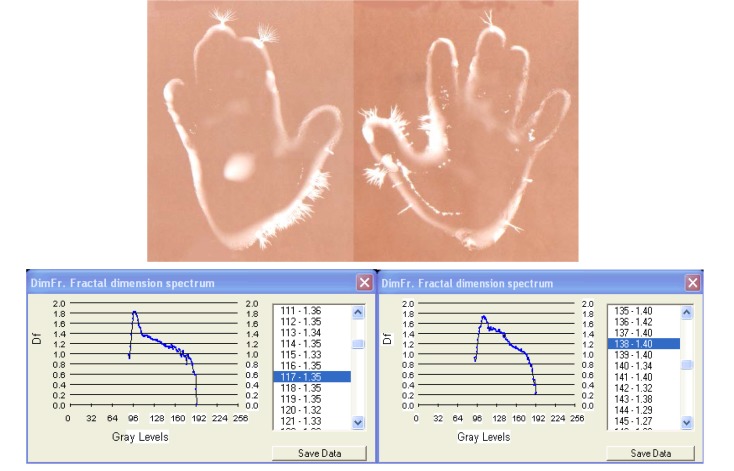
EG images with fractal dimension spectrum of both hands of subject C.T. , 14 years old, ***deaf and dumb-witness sample for harmonious acoustic stimulus***.

**Fig. 8b:**
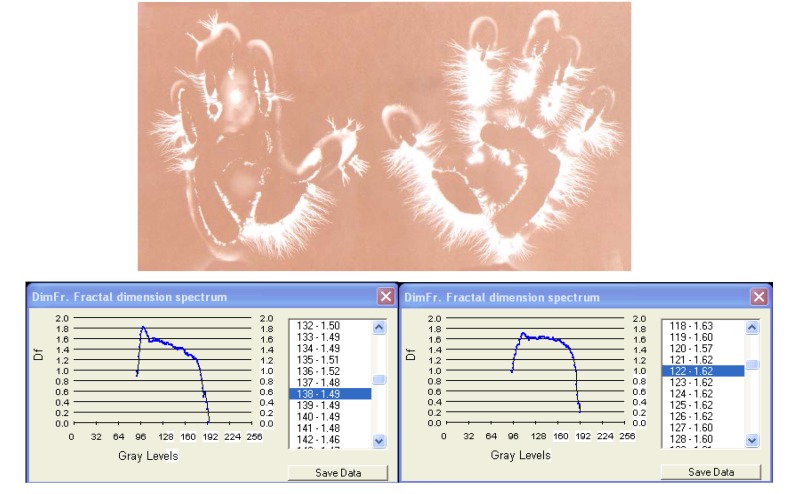
EG images with fractal dimension spectrum of both hands of subject C.T. , 14 years old - ***deaf and dumb***, immediately after harmonious sonorous stimuli – ***10 minutes***.

**Fig. 9a:**
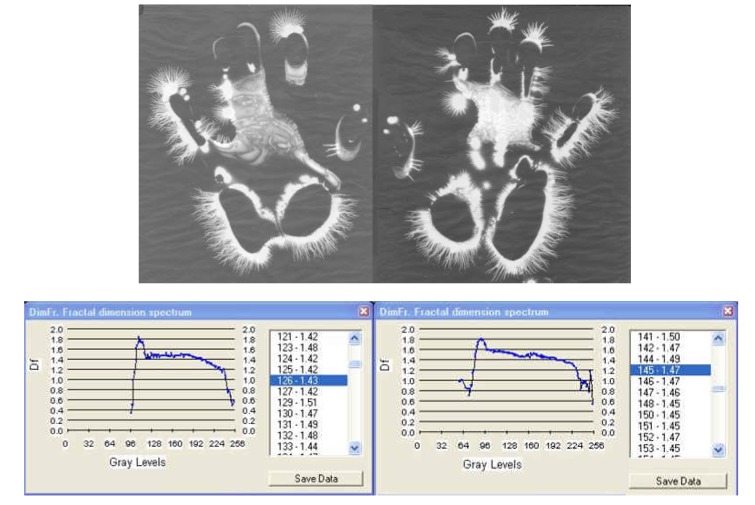
EG images with fractal dimension spectrum of both hands of subject I.V. , 44 years old, with ***binocular atrophy – witness sample for exposure to light.***

**Fig. 9b:**
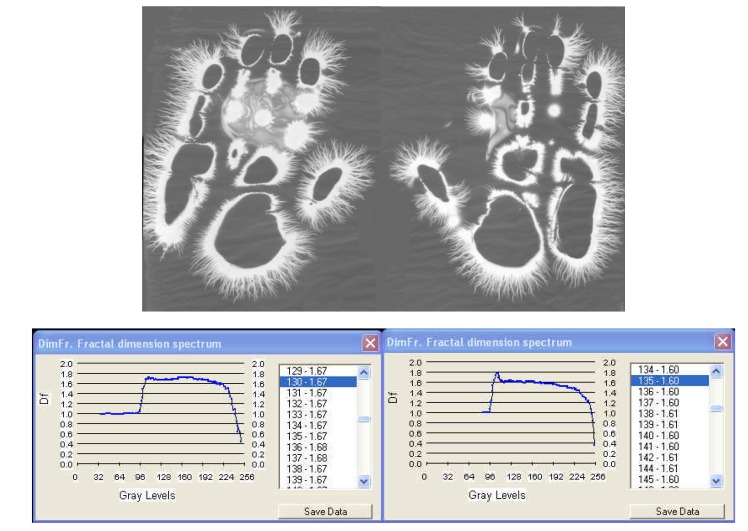
EG images with fractal dimension spectrum of both hands of subject I.V. , 44 years old, with ***binocular atrophy after exposure to blue light – 10 minutes.***

**Table 1:** Variation of *average fractal dimensions* * of EG images for the selected casuistics..

Investigation No.	Subject	State of health / Diagnosis / Testing conditions	Average fractal dimension (Df.) left/right hand
1.	L.G. **2008**	Witness for stability in time	1.58 / 1.60
2.	L.G.**1984**	Witness for stability in time	1.60 / 1.60
3.	L.G.**1978**	Witness for stability in time	1.58 / 1.56
			
1	L.G.	Witness for sonorous stress	1.43 / 1.60
2	L.G.	Sharp sonorous signal ***3 seconds***	1.39 / 1.48
3	L.G.	Sharp sonorous signal ***3 seconds***	1.28 / 1.24
			
1		Binocular atrophy	1.43 /1.47
2	I.V.	Binocular atrophy with exposure ***to blue light 10 min.***	1.67 /1.60
			
1	C.M. - 16	Deaf-dumbness	1.29/1.35
2	C.M. - 16	Deaf-dumbness with exposure ***to harmonious sounds 15 min.***	1.40/1.56
			
1	D.T.- 45	Bronchial asthma	1.51 /1.38
2		Spasmophilia	1.40 /1.43
3	A.L. - 55	Neurotic syndrome	1.49 /1.47
4	N.M. - 42	Main-Charcot Disorder	1.63 /1.61
5	I.C. -27	Form-fruste hemiparesis with aaaphasic disorders	1.64 /1.63
6	A.F. - 32	Renal insufficiency	1.60 /1.47
7	A.A. -36	Sterility, amenorrhea, long time smoker	1.59 /1.66
*The selected fractal dimension is an average on the flattened intervals in the analysed spectra, where the fractal character is preponderant.			

Processing by means of fractal analysis method, as we intend to demonstrate, brings about valuable characterization and evaluation possibilities of the multitude of data and information contained in the EG image.

We will further discuss the experimental results, focusing on the four **important study directions** suggested:

I. The images from figures a, b, c. have been selected from a longitudinal study carried out on a single subject, over a period of 30 years. The comparative analysis of the individual with himself and as a standard series versus the others, demonstrates that there is a pattern (a model that can be identified in about 75% of the entire investigations). In time variable elements occur, some of them becoming permanent and enriching the individual’s characteristic image [**4**]. This type of investigation may be constituted in a **witness series** of EG images, characteristic of each individual’s ontogenesis. It has been found that the spectrum of the fractal dimension has a recognizable (similar) shape for the three moments during the 30 years, and the average of fractal dimension varies only by 4%.

II. With **Fig. 7a**, **b**, **c** EG images taken while studying aspects of the reactivity of the human organism to auditory stimuli at palmar level: the response of the organism to non-harmonius sounds – sharp whistling with intermittence. One can notice a rapid change in time (palmar reaction at only 30 seconds from the sharp sound) and a modification of the symmetry and density of streamers, which after 3 minutes disappear almost completely, leaving only an intense luminescence unevenly distributed on the palms. The EG investigation may be used under this aspect in order to monitor certain psychophysiological and pathological processes, alongside with the current paraclinical investigations with a view to increasing the efficiency of the medical diagnosis. The fractal dimensions spectrum varies significantly during the investigation and so does the average of fractal dimensions, which opens the perspective of appreciations and even quantitative evaluations.

III. In case of **Fig. 8a**, **b** and **9a**, **b** we have considered two extreme aspects of sensorial defficiency, with special medical and anthropological relevance: the reaction of certain deaf and dumb persons to harmonious sounds and the reaction of blind people to coloured light. In both situations we observed that EG images were significantly modified by increasing of streamer density and intensity, the images becoming characteristic of the „normality” range. The fractal dimension spectrum varies significantly during the investigation and the fractal dimension average increases, like in the previous case, which clearly demonstrates the capacity of the organism, as a whole, to receive the acoustic and visual information in absence of the specialised organs of sense. It is worth mentioning the quality of the EG method of „catching” totally new, hidden effects. Therefore the EG method may be studied in certain complex processes, difficult to follow by other methods.

IV. Out of over 10,000 subjects we have studied, both healthy and sick, we have presented in **Table 1** ***average values of fractal dimension*** for the examples analysed in this paper. For the sake of comparison and as an urge to reflection to future researches we have also added the calculations for several diagnosed cases selected from our casuistics. Average fractal dimension being expressed by means of a decimal up to hundredth 1.00… enables a very fine differentiation between the values corresponding to different categories of pathologies in point of EG.

## Preliminary conclusions

Our intention in this work if to familiarize anyone interested in a universe of information revealed by fractal shapes shown in the electrographic images, in order to understand the informational and integrating adaptive function of electromagnetic fields of the human organism, having as interface, i.e intermediated by human tegument. [4,18] (informational adaptation understood as the existence of a permanent tendency to **adapt via shapes**, obtained in evolution and used to survive, alongside with the substantial and energetic processes already known nowdays. The analysis and systematization of palmar and plantar images have led to the conclusion that there are EG print peculiarities, given by a specific organization of shapes, characteristic to an individual [3,4,6]. The EG images indicate the existence of a close correlation between the electrographic print and the shapes of dermatoglyphic print (streamer extremities come out of of the pores of dermatoglyphic tracks).

Electrography analysed by means of the methods of complexity sciences, such as the fractal method, points to new aspects of our health/illness condition introducing the concept of adaptability degree –** health / adaptability / illness** which may be assessed quantitatively by means of time units and comparatively, by deviating the shape from the archetypal, primary, encoded pattern of the investigated phenomenon. In medical and anthropological thinking we currently operate with the relations ***structure/function, shape/structure***. A step forward towards refining the understanding of the vital interdependence of the human being on the complex environment of its existence would be sensing the interdependence ***structure/shape/function…as an iterative*** cycle for medical-anthropological thinking, reflecting a law of informational evolutive adaptation. Fractal shapes experienced by means of the analysed EG method are such an application.
